# Role of Carboxyl and Amine Termination on a Boron-Doped Diamond Solution Gate Field Effect Transistor (SGFET) for pH Sensing

**DOI:** 10.3390/s18072178

**Published:** 2018-07-06

**Authors:** Shaili Falina, Sora Kawai, Nobutaka Oi, Hayate Yamano, Taisuke Kageura, Evi Suaebah, Masafumi Inaba, Yukihiro Shintani, Mohd Syamsul, Hiroshi Kawarada

**Affiliations:** 1 Faculty of Science and Engineering, Waseda University, Tokyo 169-8555, Japan; sora.11-11@asagi.waseda.jp (S.K.); n.ooi.9.12@ruri.waseda.jp (N.O.); hayate.yamano@gmail.com (H.Y.); tai0723@fuji.waseda.jp (T.K.); evisuaebah@gmail.com (E.S.); inaba.masafumi@imass.nagoya-u.ac.jp (M.I.); shintani@toki.waseda.jp (Y.S.); naysriq@asagi.waseda.jp (M.S.); kawarada@waseda.jp (H.K.); 2 Institute of Materials and Systems for Sustainability, Nagoya University, Furo-Cho, Chikusa-ku, Nagoya 464-8603, Japan; 3 Research and Development Department/Innovation Center, MK-Headquarters, Yokogawa Electric Corporation, Tokyo 180-8750, Japan; 4 The Kagami Memorial Laboratory for Materials Science and Technology, Waseda University, 2-8-26 Nishiwaseda, Shinjuku, Tokyo 169-0051, Japan

**Keywords:** boron-doped diamond, carboxyl termination, amine termination, pH sensitivity, polycrystalline diamond, electrolyte-solution-gate field-effect-transistor

## Abstract

In this paper, we report on the effect of carboxyl- and amine terminations on a boron-doped diamond surface (BDD) in relation to pH sensitivity. Carboxyl termination was achieved by anodization oxidation in Carmody buffer solution (pH 7). The carboxyl-terminated diamond surface was exposed to nitrogen radicals to generate an amine-terminated surface. The pH sensitivity of the carboxyl- and amine-terminated surfaces was measured from pH 2 to pH 12. The pH sensitivities of the carboxyl-terminated surface at low and high pH are 45 and 3 mV/pH, respectively. The pH sensitivity after amine termination is significantly higher—the pH sensitivities at low and high pH are 65 and 24 mV/pH, respectively. We find that the negatively-charged surface properties of the carboxyl-terminated surface due to ionization of –COOH causes very low pH detection in the high pH region (pH 7–12). In the case of the amine-terminated surface, the surface properties are interchangeable in both acidic and basic solutions; therefore, we observed pH detection at both low and high pH regions. The results presented here may provide molecular-level understanding of surface properties with charged ions in pH solutions. The understanding of these surface terminations on BDD substrate may be useful to design diamond-based biosensors.

## 1. Introduction

pH sensors play a crucial role in biological processes, the food industry, and environmental and domestic applications. pH sensing is commonly performed by glass membranes and it has been established for many years and widely used in laboratories and manufacturing processes. Despite its popular use in various fields, traditional glass membrane pH sensors has disadvantages, such as fragility and large size [1]. These drawbacks of glass membrane pH sensors have motivated the development of solid state field-effect transistor (FET) pH sensors. Various FET pH sensors based on different technologies and materials have been reported. Replacement of the glass membrane pH sensor with a FET pH sensor enables the possibilities of miniaturization and sensitivity improvement. The FET pH sensor concept was introduced in 1972 using silicon dioxide (SiO_2_) as a pH-sensitive dielectric [2]. To obtain higher sensitivity, researchers subsequently investigated other dielectric materials, such as Al_2_O_3_ [3], Si_3_N_4_ [4], Ta_2_O_5_ [5], and SnO_2_ [6]. Even though ion-selective FETs (ISFETs) show Nernst or near-Nernst responses (regardless of the material used for the dielectric), ISFETs have disadvantages. For example, ISFETs generally exhibit hysteresis and the drift effect, which leads to measurement error owing to the unreliability of the passivation layer in aqueous media [7]. In addition, ISFET-based pH sensors also show a slow response because of their thick passivation layers [8]. Due to the drawbacks, a robust material is in demand to improve the performances of the previous FET pH sensor. Boron-doped diamond (BDD) brings new prospects as a substrate for pH sensors. 

BDD has attracted much interest in biosensor research. Owing to its excellent properties, such as a wide potential window [9], biocompatibility [10], chemical and physical stability [11], and the ability for surface termination [12], BDD is an ideal substrate for integrated sensing and signal processing. Furthermore, BDD is also commercially available, relatively cheap, and easy to prepare in the laboratory. BDD can be synthesized by chemical vapor deposition (CVD) using boron and methane gas, or the ion implantation technique. Considering the robustness and reliability features for succeeding pH sensors, the diamond solution-gate field effect transistor (SGFET) was introduced [13] as a replacement to the ISFET pH sensor using un-doped two-dimensional hole gas (2DHG) diamond substrate. The structure of the diamond SGFET is analogous to an ISFET except it does not require a passivation layer, which allows the channel surface to be directly exposed to the electrolyte solution. Owing to the simplicity of the fabrication of diamond SGFETs, they have advantages over ISFET-based pH sensors, such as low-risk measurement error [14] and low noise [15]. Other advantages of using diamond SGFETs for pH sensors are the possibilities of small-scale pH sensors, reliable pH detection because the diamond substrate has good stability properties, easy enhancement of the pH sensitivity by surface modification, and diamond SGFET pH sensors are robust compared with other solid-state pH sensors owing to the physical hardness and chemical inertness of diamond. 

As aforementioned, pH sensitivity of the diamond pH sensor can be enhanced by surface modification. The past few decades have seen rapid advancement in surface modification methods and surface functionalization of different functional groups on the diamond SGFET channel surface. There are numerous reports of surface functionalization of diamond SGFETs for pH sensor applications via modification with different functional groups, such as hydrogen [16], oxygen [17], fluorine [18], and amine [19]. However, the pH sensitivity of the surface-bound functional groups, such as amine and carboxyl groups, has not been comprehensively studied. For instance, in our previous study on amine termination of a diamond SGFET [19], we mainly focused on the relationship between ultraviolet irradiation and the site-binding coverage. In the present work, we focus on understanding the near-surface mechanism of NH_2_/NH_3_^+^ binding on the BDD SGFET surface in different pH solutions. In addition, reports on the pH sensitivity of carboxyl termination are rare. The main objectives of the present study are to describes near-surface mechanism of carboxyl and amine termination on BDD channel surfaces and determine how the pH sensitivity of the surface-modified BDD (ultimately, the surface-bound functional groups) is affected in different pH solutions. We attempt to establish the process for carboxyl termination on the BDD SGFET surface by controlled anodic oxidation. We also demonstrate amine termination by nitrogen radicals (N-radicals) from a nitridation process. The current-voltage (*I-V*) characteristics of the BDD SGFET and the relationship between the *I-V* characteristics and the controlled anodic oxidation process using sequential increment of voltage are discussed in detail. Finally, the pH sensitivities of the carboxyl- and amine-terminated BDD SGFET channel surfaces in the acidic/basic regions are compared. The understanding of these surface properties can be useful to design diamond-based sensors for detection of charged particles or biomolecules.

## 2. Materials and Methods

### 2.1. Thin Film Boron-Doped Layer Deposition

First, a 5 mm × 5 mm diamond substrate was immersed in a mixture of sulfuric acid and nitric acid at 240 °C for 1 h to remove residual grime and oil from the manufacturing process. A thin boron layer was then deposited on the diamond substrate by microwave plasma-assisted (CVD) system (ASTeX Corp., Wilmington, MA, USA). Trimethylboron gas was used as the doping gas at a flow rate 0.9 standard cubic centimeters per minute (sccm). Hydrogen (H_2_) was used as the carrier gas and methane (CH_4_) was used as the carbon source, at gas flows rates of 300 sccm and 0.9 sccm, respectively. The reactor was maintained at 50 Torr and power of 1200 W was applied. The deposition time was 5 min and the substrate temperature was controlled to be approximately 750 °C, as measured by a radiation thermometer. The substrate was cooled in the reactor for 20 min under 100 sccm hydrogen gas flow. To evaluate the conductivity of the diamond surface after thin boron film deposition, we performed direct current Hall measurements by the van der Pauw method with a DC Hall system model HL5500 (Nanometrics, Milpitas, CA, USA). The p-type carrier concentration was on the order of 10^13^ cm^−2^/square and the sheet resistivity was 10–30 kΩ.

### 2.2. SGFET Fabrication

**Figure 1:**
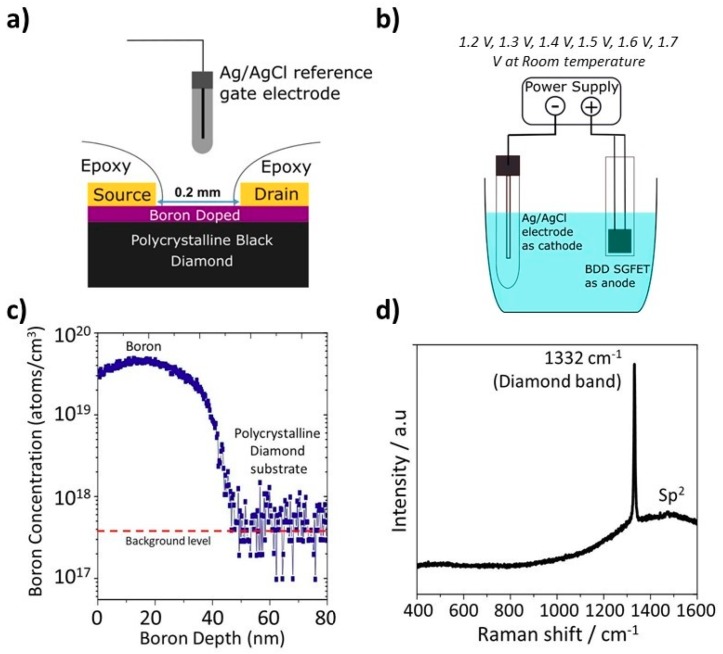
(**a**) Schematic diagram of the cross-section of the BDD SGFET and (**b**) a diagram the anodic oxidation process. (**c**) Boron doping profile of a diamond substrate. (**d**) Raman spectra of BDD substrate. The excitation wavelength is 633 nm.

The SGFET fabrication process started with evaporation of pure gold onto the substrate surface by an electron beam evaporator. A thin metal mask was placed at the center of the BDD substrate, while the uncovered parts of the substrate were exposed to gold evaporation to form symmetrical drain and source electrode pads. The thickness of the gold layer was 150 nm. The unexposed area formed a channel region, with a width of 5 mm and a length of 0.2 mm. The BDD substrate was set on a glass slide with transparent adhesive and a pair of wires was connected to the source and drain contact pads with conductive silver paste (Chemtronics, Kennesaw, GA, USA) to enable an external bias. The conductive paste was allowed to dry before the source and drain contact pads were encapsulated with epoxy resin Araldite 2000 (Huntsman Advanced Materials, Basel, Switzerland). After encapsulation only the channel region was exposed to the electrolyte solution. Figure 1a shows a schematic of the cross-section of the BDD SGFET device with an Ag/AgCl reference gate electrode used to supply the gate bias.

### 2.3. Surface Modifications

#### 2.3.1. Anodic Oxidation

The BDD SGFET was anodized in 100 mL buffer solution (pH 7) using a two-electrode configuration, with the BDD SGFET as the anode and an Ag/AgCl electrode as the cathode (Figure 1b). The same potential was applied to both the BDD SGFET source and drain. Anodic oxidation was performed at anodization voltages of 1.2, 1.3, 1.4, 1.5, 1.6, and 1.7 V at room temperature. A semiconductor device parameter analyzer model B1500A (Keysight, Tokyo, Japan) with a custom LabVIEW program (National Instruments, Austin, TX, USA) was used to supply the current and voltage. Anodic oxidation was initiated with sweeping potentials from 0 to 1.2 V, and the voltage was swept back to 0 V in decrements of 20 mV for 5 s to complete a full scan. Anodic oxidation was repeated on the same device by varying the sweeping bias from 1.2 to 1.7 V. After each anodic oxidation scan, the transfer curve of the BDD SGFET was measured in pH 7 electrolyte to determine the threshold voltage (V_TH_) shift as an indication of the oxidation process.

#### 2.3.2. Nitrogen and Amine Termination

The oxygen-terminated channel surface BDD SGFET was exposed to radio frequency (RF) nitrogen radicals in ultra-high vacuum molecular beam epitaxy (MBE) of III-nitride (ARIOS, Tokyo, Japan). A nitrogen-hydrogen gas mixture 96%:4%, was introduced into the RF plasma with a nitrogen gas flow rate of 2.0 sccm. The purpose of introducing a small amount of hydrogen into the chamber during the nitridation process was to clean the substrate surface and to terminate any surplus dangling bonds and form an amine-terminated surface. The chamber pressure was maintained at 5 × 10^−3^ Pa during the nitridation process. The channel surface of the BDD SGFET was exposed to RF nitrogen plasma for 20 min at room temperature. 

### 2.4. SGFET I-V Characterization

The current and voltage characteristics of the BDD SGFETs were measured with the semiconductor device parameter analyzer model B1500A (Keysight, Tokyo, Japan) with a custom LabVIEW program (National Instruments, Austin, TX, USA) to supply a constant current to the drain and gate electrodes. A commercial Ag/AgCl reference electrode was used to apply the gate voltage. SGFET current and voltage characterization was performed at room temperature in Carmody buffer solutions in the pH range of 2–12. The Carmody buffer solutions consisted of mixed solutions of boric acid (BH_3_O_3_), citric acid monohydrate (C_6_H_8_O_7_), and trisodium phosphate dodecahydrate (Na_3_PO_4_·12H_2_O). The pH of each solution was adjusted and measured with a digital pH meter model PH72 (Yokogawa Electric Corp., Tokyo, Japan). Calibration of the digital pH meter was performed using two-point calibration in phthalate pH standard solution (pH values 6.86 ± 0.01 and 4.01 ± 0.01). All the chemicals were purchased from Kanto Chemical Co., Inc. (Tokyo, Japan).

## 3. Results and Discussion

### 3.1. BDD SGFET Direct-Current Hall Measurement Profile, I-V Characteristics, and Precise V_TH_ Control by Partial Oxidation

**Figure 2:**
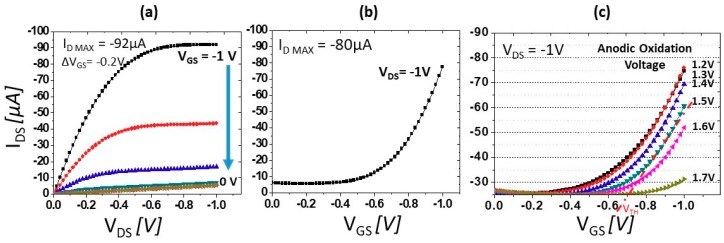
I-V characteristics of the BDD SGFET: (**a**) I_DS_-V_DS_; (**b**) I_DS_-V_GS_; and (**c**) V_TH_ shift of the BDD SGFET after anodic oxidation with voltage scans of 1.2 to 1.7 at steps of 0.1V applied sequentially.

The conductivity of the BDD substrate, measured by the Hall system, gives a sheet resistance on the order of 10 kΩ per square, and the carrier concentration is on the order of 10^13^ cm^−2^. The BDD substrate has low mobility (<10 cm^2^ V^−1^ s^−1^) due to ionized boron as an impurity [20], although this is adequate for fabrication and operation of a SGFET. The boron-doping profile obtained by secondary ion mass spectroscopy (SIMS) model Atomika 4500 (Fibics Inc., Ottawa, ON, Canada) gives a thickness of 40 nm and a boron concentration in the order of 10^19^ cm^−3^, as shown in SIMS data in Figure 1c. The surface area density estimated from the SIMS results is in the order of 4 × 10^13^ cm^−2^, which is comparable with the carrier concentration measured with the Hall system. The fraction of sp^2^ is relatively low and considered to be negligible, as estimated by Raman spectroscopy (Renishaw, Gloucestershire, UK) measurements depicted in Figure 1d. Thus, the pH sensitivity is dependent on the surface termination, contrary with the findings of previous studies [21], in which a significant sp^2^ peak exists in heavily BDD with a boron concentration in the order of 10^20^ cm^−3^ and may affect the performance/response of BDD. Reproducibility of the BDD pH sensor is possible if we obtain the similar sheet resistivity of BDD and maintain the boron doping profile. Figure 2a shows the curves of the drain–source current (I_DS_) as a function of the source voltage (V_DS_) curves of the BDD SGFET at various V_GS_ values from 0 to −1 V. The source–drain current characteristics were measured with respect to the gate voltage (V_GS_) and drain voltage (V_DS_) in Carmody pH 7 buffer solution. The device exhibits the typical characteristics of a transistor with triode and saturation regions. The maximum current density (I_DS_ max) is −92 µA, and transconductance (gm) of 250 µS mm^−1^ is observed at V_GS_ = −1 V. The relationship between I_DS_ and V_GS_ is shown in Figure 2b. A V_TH_ value of −0.65 V at V_DS_ = −1 V is estimated for this device. 

After each anodic scan, the variations of I_DS_ with V_GS_ was also measured at V_DS_ = −1 V to determine the V_TH_ shifts of the BDD SGFET. As shown in Figure 2c, the V_TH_ shifts to a more negative potential as the scan voltage increases from 1.2 to 1.7 V. The present findings are consistent with the findings of our prior study, where we investigated the effect of ozone treatment on an un-doped diamond SGFET [22]. When the BDD SGFET channel was pre-oxidized at 1.2 V, hydrogen atoms were removed from the BDD surface, leaving unstable dangling carbon radicals. These radicals can react with water molecules in the electrolyte to form different types of oxygen species during anodic oxidation, such as hydroxyl (≡C–OH), carbonyl (=C–O), and carboxyl (–COOH) groups [23]. Consecutive anodic oxidation reduced the carrier concentration and conductivity of the BDD surface. Therefore, as oxidation of the diamond surface increases, the sheet resistance increases, and a higher gate bias is required to overcome the resistance of the BDD surface. Hence, V_TH_ shifts to a more negative potential. Oxygen termination of the BDD surface was investigated by X-ray photoelectron spectroscopy (XPS). However, no distinct differences are observed before and after anodic oxidation, because the surface is covered not only with chemisorbed oxygen species, but also with physisorbed oxygen species. Precise XPS measurements might be possible if coupled with an in situ ambient anodic oxidation process.

To produce a robust and stable pH sensor using BDD, stability tests are a crucial to ensure reusability of the pH sensor. In previous study, we investigated the stability of a BDD pH sensor after 3 and 10 months [24]. The current decreased by 15% and 18% after 3 and 10 months, respectively, owing to the channel surface resistivity linearly increasing with time. These findings show that the BDD pH sensor is stable, operates well for a long period of time, and has the potential for long-term reusability. However, to prolong the longevity of the pH sensor device, maintenance must be performed. It is recommended that the BDD SGFET pH sensor is washed with distilled water after use and dried before storing. 

### 3.2. Sensing Characteristics of Carboxyl- and Amine-Terminated BDD SGFETs

**Figure 3:**
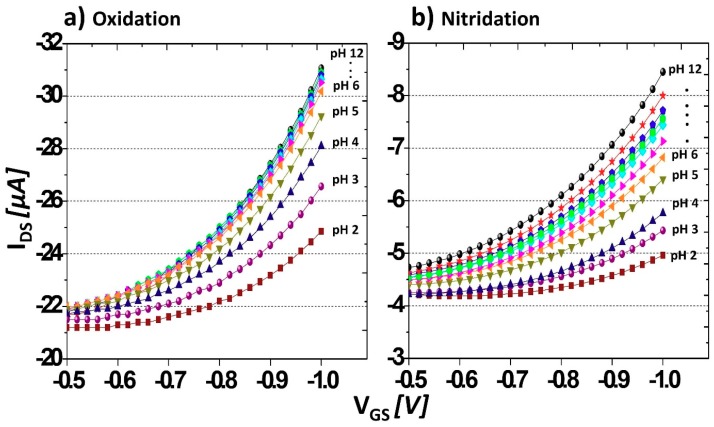
V_TH_ shift of the BDD SGFET after (**a**) anodic oxidation and (**b**) nitridation or amine termination.

Figure 3a, b show the variation of I_DS_ with V_GS_ for oxidation (or carboxyl termination) and amine termination of the BDD surface as the pH is changed. The sensitivity of the BDD SGFET to pH was investigated based on the shift of V_TH_ as the solution pH was varied. In Figure 3a, V_GS_ was swept from 0 to −1 V and V_DS_ was set at −1 V. A V_TH_ shift is observed at low pH (pH 2–6), whereas no shift is observed at high pH (pH 7–12). This suggests that most of the BDD surface was covered with –COOH groups as a result of the anodic oxidation process. Furthermore, based on the distinct device response in the acidic and basic regions owing to ionization of –COOH groups in acidic solution, it is reasonable to suggest that –COOH groups are dominant on the BDD surface. In contrast, oxygen termination by ozone treatment results in negligible changes in the pH sensitivity in the pH range of 2–12 [25]. Carboxylic acids, which have typical pKa values in the range of 2–5 [26], will ionize in aqueous media because their pKa values are lower than the pH and hydrogen ions will dissociate from the –COOH groups into the solution. Thus, at low pH values, the carboxylate ions (–COO−) act as a negative channel surface, which can attract the abundant H^+^ ions in the acidic electrolyte, resulting in depletion of hole carriers under the channel and a decrease in the device conductivity. The lower the pH, the greater the amount of H^+^ in the electrolyte, so the lower the conductance of the device and the lower the current. At high pH values (pH > 7), there is a high level of electrostatic repulsion of the hydroxide ions (−OH) from the channel surface. Thus, no shifts in the V_TH_ are observed at high pH values because fewer H^+^ ions are present in the electrolyte and fewer holes are depleted under the channel surface as the pH increases. Therefore, no changes in the current are expected in basic solutions (pH 7–12).

In the case of amine termination, V_TH_ shifts are observed in the pH range 2–12 (Figure 3b). Specifically, and in contrast to the case of carboxyl termination, significant V_TH_ shifts are detected at high pH values (pH 7–12). This suggests that amine modification of the BDD surface increases the pH sensitivity of the electrode at high pH. In a previous study [27], we showed that 50% of the diamond surface is covered with nitrogen-related bonds, as estimated by XPS from which the NH_2_ coverage cannot be determined. The other 50% of the diamond surface is covered with oxygen functional groups including carboxyl groups. The amine functionality on the BDD surface is in the form of free amine (–NH_2_) or protonated amine (–NH_3_^+^) groups, with typical pKa values in the range of 8.7–10.7 [26]. In solutions where the pH is less than the pKa value, amine groups exist as protonated amine (–NH_3_^+^) groups on the BDD surface. As more H^+^ ions bond to the amine groups to form –NH_3_^+^ on the BDD surface, many of the carrier holes are repelled from the channel surface, resulting in a low current at low pH values. In contrast, when the pH of the solution is greater than the pKa value, amine groups exist as free amine (–NH_2_), giving the BDD surface a negative electron affinity or positive sites [28]. The negative ions (–OH) in high pH solutions can readily approach the diamond surface, thereby generating a source–drain current and causing a V_TH_ shift. Compared with carboxyl termination, amine termination affords interchangeable BDD surface properties in the acidic and basic regions, which is highly advantageous for sensing in a wide pH range of 2–12. 

**Figure 4:**
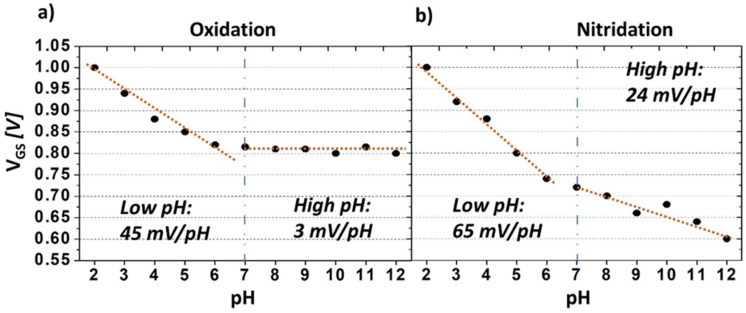
pH sensitivity with respect to the change in the gate voltage in solutions with a wide pH range of 2–12. (**a**) Oxidation or carboxyl termination, and (**b**) nitridation process or amine termination.

The V_GS_ values were calculated for pH 2–12 while keeping *I_DS_* constant. As shown in Figure 4a, following carboxyl termination by anodic oxidation, the BDD surface exhibits pH sensitivities of 45 mV/pH in low pH solutions and 3 mV/pH in high pH solutions. Following amine termination by nitridation, the BDD surface exhibits higher pH sensitivity than the carboxyl-terminated surface with sensitivities of 65 and 24 mV/pH in low and high solutions, respectively (Figure 4b). As mentioned above, the pH sensitivity of the amine-terminated surface at high pH can be attributed to the existence of –NH_2_ groups, which provide a positively-charged surface that can attract negative ions (–OH) in basic solutions. In contrast, at low pH, the sensitivity of the partially amine-terminated surface is higher than the carboxyl-terminated surface and exceeds the Nernst response of 59 mV/pH. The enhancement in the pH sensitivity in the acidic region is attributed to the presence of a combination of carboxyl and amine groups. In low pH solutions, the amine groups are protonated, while the carboxyl groups are present as carboxylate ions, which attract H^+^ ions. Both species cause greater depletion in the hole carriers and decrease the device conductance. In the basic region, the pH sensitivity is not as high as in the acidic region, which is in agreement with previous reports, which concluded that the size of the counter-ions of the phosphate-based solution is not large enough for pH detection at pH > 7 [29]. Alternatively, tetraborate-based pH solutions could be used to increase the pH sensitivity in the basic region. Despite its lower pH sensitivity across the high pH range (pH 7 to pH 12) compared with the ISFET, which has been reported to show a near Nernst slope value [30], the BDD SGFET pH sensor exhibits good reproducibility and accurate pH sensitivity data [24]. 

In an earlier report, deprotonation/protonation of –COOH and NH_2_ groups changed the surface characteristics from hydrophobic to hydrophilic, or vice versa [31]. The carboxyl-terminated surface was characterized as hydrophobic at low pH and hydrophilic at high pH, whereas the amine-terminated surface was hydrophilic at low pH and hydrophobic at high pH.

The carboxyl groups are gradually deprotonated when the pH changes from pH 2 to pH 12. As the proportion of carboxylate ions increases, the surface develops into a more hydrophilic surface. In the present study, pH dependence is observed at low pH because the carboxyl-terminated diamond surface is hydrophobic, which prevents adsorption of water molecules, so there is a higher chance of H^+^ ions being attracted to the diamond channel surface. As the pH increases, the surface becomes more charged as the –COOH functional groups deprotonate to –COO^−^, creating a hydrophilic surface. For pH > 7, ionization of –COOH to –COO^−^ gives an ionic center that can interact with water molecules to form a hydrate layer, which prevents the hydroxide ions (–OH^−^) in solution from approaching the diamond surface, so no ion detection is observed at high pH. In contrast to carboxyl-terminated surface, the amine-terminated surface is hydrophilic at low pH (pH 2–6) and hydrophobic at high pH (pH 7–12) because of the protonation and deprotonation of amine groups, respectively. At low pH, even though the amine-terminated surface is hydrophilic, however the solubility of the amine groups in solution is lower than that of carboxyl functional groups, so not all of the amine groups form hydrogen bonds with water molecules. Most of the amine groups were protonated to –NH_3_^+^, causing hole repulsion from the channel surface, while at high pH (pH 7–12), the amine groups exist as free amine (NH_2_) groups, carry positive charges producing a hydrophobic surface. Since the surface is hydrophobic, it does not form hydrogen bonds with water molecules, and hydroxide ions (–OH^−^) are attracted to the BDD surface. Therefore, the amine-terminated surface shows the pH sensitivity in basic solution.

## 4. Conclusions

We have shown the near-surface mechanism of carboxyl and amine termination and how it affects the pH sensitivity of the BDD surface. Our findings show that anodic oxidation produces carboxyl groups that are deprotonated to form negatively-charged carboxylate ions, which only attract positive ions. Thus, the apparent pH sensitivity in the low pH region (2–6) is attributed to the abundant H^+^ ions in the acidic solution, whereas the pH sensitivity is poorer in the high pH region (7–12). In contrast, the nitridation process generates amine groups on the BDD channel surface. The ionization properties of the amine groups are dependent on the pH. pH sensitivity of the amine-terminated surface is observed in both the acidic and basic regions. The present findings enhance the understanding of the role of carboxyl termination and amine termination in relation to the pH sensitivity of the BDD substrate.
